# Early Monitoring Antiangiogenesis Treatment Response of Sunitinib in U87MG Tumor Xenograft by ^18^F-FLT MicroPET/CT Imaging

**DOI:** 10.1155/2014/218578

**Published:** 2014-04-09

**Authors:** Xiao Bao, Ming-Wei Wang, Yong-Ping Zhang, Ying-Jian Zhang

**Affiliations:** ^1^Department of Nuclear Medicine, Fudan University Shanghai Cancer Center, Shanghai 200032, China; ^2^Department of Oncology, Shanghai Medical College, Fudan University, Shanghai 200032, China; ^3^Center for Biomedical Imaging, Fudan University, Shanghai 200032, China

## Abstract

*Aim*. It was aimed to monitor early treatment response of Sunitinib in U87MG models mimicking glioblastoma multiforme by longitudinal ^18^F-FLT microPET/CT imaging in this study. *Methods*. U87MG tumor mice were intragastrically injected with Sunitinib at a dose of 80 mg/kg for consecutive 7 days. ^18^F-FLT microPET/CT scans were acquired on days 0, 1, 3, 7, and 13 after therapy. Tumor sizes and body weight were measured. Tumor samples were collected for immunohistochemical analysis of proliferation and microvessel density (MVD) with anti-Ki67 and anti-CD31, respectively. *Results*. The uptake ratios of tumor to the contralateral muscle (T/M) of ^18^F-FLT in the Sunitinib group decreased from baseline to day 3 (T/M_0_ = 2.98 ± 0.33; T/M_3_ = 2.23 ± 0.36; *P* < 0.001), reached the bottom on day 7 (T/M_7_ = 1.96 ± 0.35; *P* < 0.001), and then recovered on day 13. The T/M of ^18^F-FLT uptake in the control group remained around 3.0. There was no difference for the tumor size between both groups until day 11. ^18^F-FLT uptakes of tumor were correlated with Ki67 staining index and MVD. *Conclusion*. Early therapy response to Sunitinib could be predicted via ^18^F-FLT PET, which will contribute to monitoring antiangiogenesis treatment.

## 1. Introduction

Angiogenesis is a fundamental physiological process to form new blood vessel to support cancer growth and development by providing nutrients and oxygen. It occurs for almost all solid tumors such as glioblastoma (GBM) and thus antiangiogenesis therapeutics are increasingly applied to treat various cancers. GBM is the most aggressive primary malignant brain tumor in humans with a 5-year survival rate under 5% and median overall survival of only 12–14 months [1]. GBM features rich vascularization due to the high expression of various proangiogenic factors, which makes antiangiogenesis as an attractively newly emerging targeted therapy strategy of GBM, although the standard treatments of GBM are still surgical operation, radiotherapy, and chemotherapy at present [2]. For instance, vascular endothelial growth factor inhibitor, bevacizumab, has been the sole antiangiogenesis targeted therapeutic licensed by the FDA for use in GBM [3]. In order to discover more effective anticancer agents, multitargeted tyrosine kinase inhibitors (TKIs), such as Sunitinib, are being under clinical investigations owing to their antitumor capabilities via the pathways of antiangiogenesis as well as antiproliferation [4]. Sunitinib as antiangiogenic therapeutic has already been used to treat renal carcinoma, gastrointestinal stromal tumors, lung cancer, and other solid tumors [5, 6]; however, it showed controversial value in primary or recurrent GBM therapy [7–9]. Moreover, antiangiogenic treatment faces currently some other challenges, such as low objective response rate and a huge economic burden of the high price [4, 10]. Since TKIs are predominant cytostatic therapeutics rather than cytotoxic therapeutics, decrease in tumor size caused by TKIs therapy might take 3–6 months or might not always occur [11, 12]. As results, conventional methods relying on tumor size changes, that is, response evaluation criteria in solid tumors (RECIST) is insufficient and even inappropriate for the response evaluation by monitoring the variation of tumor size after targeted therapies. Notably and importantly, size-based treatment evaluation is unable to discriminate residual viable tumor tissue from fibrosis. In contrast, molecular imaging by PET/CT with specific functional probes can visualize and evaluate biological and metabolic activity status of tumor cells [13]. So far, ^18^F-FDG PET/CT as the core role of PET response evaluation criteria in solid tumors (PERCIST) has been successfully used for monitoring early response of cytotoxic chemotherapy. However, it showed some limitations for ^18^F-FDG PET/CT to follow the therapy efficacy of antiangiogenesis due to the nonspecific uptake in benign tissues [14–16]. Therefore, it is in great demand to develop an alternative molecular imaging method using suitable PET probes for the early effective therapy evaluation of targeted treatment.

Beyond ^18^F-FDG, 3′-deoxy-3′-^18^F-fluorothymidine (^18^F-FLT) is another widely used PET molecular imaging probe in preclinical and clinical studies and has presented its superiority to predict early therapeutic response of cancers due to its unique cell uptake mechanism involved in DNA synthesis pathway via thymidine kinase-1. ^18^F-FLT is a radiolabeled nucleoside analogue and is generally used to image cell proliferation by PET/CT for tumor detection and grade. Moreover, previous studies had already shown that ^18^F-FLT PET/CT played a clinically useful role for predicting treatment response of cytotoxic chemotherapy and radiotherapy [17–20]. However, ^18^F-FLT PET/CT as response biomarker for cytostatic therapeutics of antiangiogenesis has not been fully proved and is still without universal understanding according to current publications [14, 15]. Consequently, more efforts are needed to further confirm the potential of ^18^F-FLT PET/CT in monitoring early response to antiangiogenic agents [21]. In the current study, we proposed to investigate the feasibility of ^18^F-FLT PET/CT to monitor early treatment response of antiangiogenesis via Sunitinib in a xenograft U87MG tumor model mimicking GBM.

## 2. Materials and Methods

### 2.1. Cell Lines and Tumor Models

The human glioblastoma multiforme cell line U87MG was purchased from Cell Bank, Shanghai Institutes for Biological Sciences, Chinese Academy of Sciences, and grown in DMEM medium (Gibco) supplemented with 10% fetal bovine serum and 1% penicillin/streptomycin (P/S) under a humidified 5% CO_2_ atmosphere at 37°C. The cells were collected by trypsinization with 0.25% trypsin/EDTA. Female athymic Balb/c nude mice (4–6 weeks) were obtained from Department of Laboratory Animal Science, Fudan University, and allowed to acclimatize for one week in the animal facility before any intervention was initiated. The U87MG tumor model was generated by subcutaneous injection of 5 × 10^6^ tumor cells in the right shoulders of the mice. Caliper measurements of perpendicular axes of the tumor were performed to follow up tumor growth. The mice weight was also measured. The treatment was initiated when the tumor reached a diameter between 8.0 and 12.0 mm (3-4 weeks after inoculation).

### 2.2. Experimental Design

Table 1 demonstrated the experimental design in this study. Mice were randomized into two major groups: the imaging group (*n* = 10) and the immunohistochemical (IHC) staining group (*n* = 27). The main purpose of using additional group mice for the IHC staining was to ensure the accuracy of the data in IHC group and not disrupt the consistency in the imaging group. Each major group was then divided into Sunitinib treatment group and control group. The treatment group was intragastrically administrated with Sunitinib (Dalian Melone Pharmaceutical Co., Ltd.) at a dose of 80 mg/kg for consecutive 7 days while the control group received oral administration of vehicle alone. Sunitinib was suspended in carboxymethylcellulose (CMC) solution (CMC 5%, NaCl 1.8%, Tween 80 0.4%, and benzyl alcohol 0.9% in distillated water). The imaging group was scanned with ^18^F-FLT microPET/CT on days 0, 1, 3, 7, and 13 after therapy initiation. Mice in the IHC staining group were sacrificed on corresponding imaging time points for IHC analysis. There were up to 3 mice sacrificed in Sunitinib and control groups, respectively, on each time point. Tumor dimensions and mice body weight were measured every other day to follow up tumor growth. The tumor volume was calculated from the following formula: tumor volume = *a* × (*b* ^2^)/2, where *a* and *b* represent the tumor length and width, respectively.

### 2.3. MicroPET/CT Imaging

MicroPET/CT scans and image analysis were performed using an Inveon microPET/CT (Siemens Medical Solution). Each U87MG tumor-bearing mouse was injected with 11.1 MBq (300 *μ*Ci) of ^18^F-FLT via tail vein. Ten-minute static scans were acquired at 1.0 h after injection and animals were maintained under isoflurane anesthesia during scanning period. The images were reconstructed using three-dimensional ordered-subset expectation maximization (OSEM3D)/maximum algorithm. For each microPET/CT scan, 4.0 mm diameter spherical regions of interest (ROIs) were drawn over both the tumor and the contralateral muscle on decay-corrected images using Inveon Research Workplace to obtain percentage injected dose per gram (%ID/g) and standardized uptake values (SUV). The highest uptake point of entire tumor was included in ROI and no necrosis area was allowed. The mean %ID/g (%ID/g_mean_), maximal %ID/g (%ID/g_max⁡_), mean SUV (SUV_mean_), and maximal SUV (SUV_max⁡_) were measured. Additionally, the ratio of %ID/g_max⁡_ of tumor to the contralateral muscle (T/M) was calculated.

### 2.4. Immunohistochemistry and Histology

On days 0, 1, 3, 7, and 13 after therapy initiation, in order to minish the sampling error of IHC staining, three U87MG tumor-bearing mice in each group were sacrificed and tumor samples were collected to fix in 10% formalin neutral buffer solution for paraffin embedding. Paraffin-embedded tissues were cut into 4 *μ*m sections and stained with mouse anti-human Ki67 antibody (1 : 100, Abcam) and rat anti-mouse anti-CD31 (Abcam). Endogenous peroxidase activity was blocked with 3% H_2_O_2_ for 15 min. Antigen retrieval was performed by boiling the sections for 10 min in citrate buffer (pH 6.0) and cooling at room temperature, followed by blocking with 10% normal goat serum for 1.0 h. The sections were incubated with optimal dilutions of anti-Ki67 and anti-CD31 overnight at 4°C, and then Ki67^+^ cells and CD31^+^ areas were detected with horseradish peroxidase- (HRP-) conjugated anti-mouse/rat secondary antibodies using an EnViSion Detection kit (Gene Tech Co., Ltd., Shanghai, China). After washing with PBS three times for 5.0 min each time, the immune complexes were visualized using a Peroxidase Substrate DAB kit (Gene Tech Co., Ltd., Shanghai, China) according to the manufacturer's instructions. Finally, the slices were counterstained with hematoxylin and dehydrated. Ki67^+^ cells and CD31^+^ areas were counted on 4 randomly selected visual fields per section of each sample under high power.

### 2.5. Immunohistochemistry Analysis

Adobe Photoshop CS5 software was used to assess the total number of Ki67-positive cells and CD31-positive vessels. The Ki67 staining index (SI) was defined as the percentage of positive nuclei in relation to the total number of nuclei. CD31-positive vessels counting method was modified from the protocol described by Weidner et al. [22]. Microvessel density (MVD) was assessed by light microscopy in areas containing the highest numbers of CD31-positive vessels per area (neovascular “hot spots”) [22]. All stained endothelial cells or cell clusters were counted as one microvessel. When two or more positive foci seemed to belong to a single continuous vessel, they were counted as one microvessel. Vessel lumens were not essential. For each section, individual microvessel counts were made on four randomly high-powered fields at 200x magnification. MVD count was defined as the average of the vessel numbers on the 4 fields.

### 2.6. Statistical Analysis

Quantitative data were presented as mean ± SD. One way analysis of variance was used to compare groups of two by SPSS 16.0. *P* values <0.05 were considered statistically significant.

## 3. Results

### 3.1. Sunitinib Treatment Inhibited U87MG Tumor Growth

**Figure 1:**
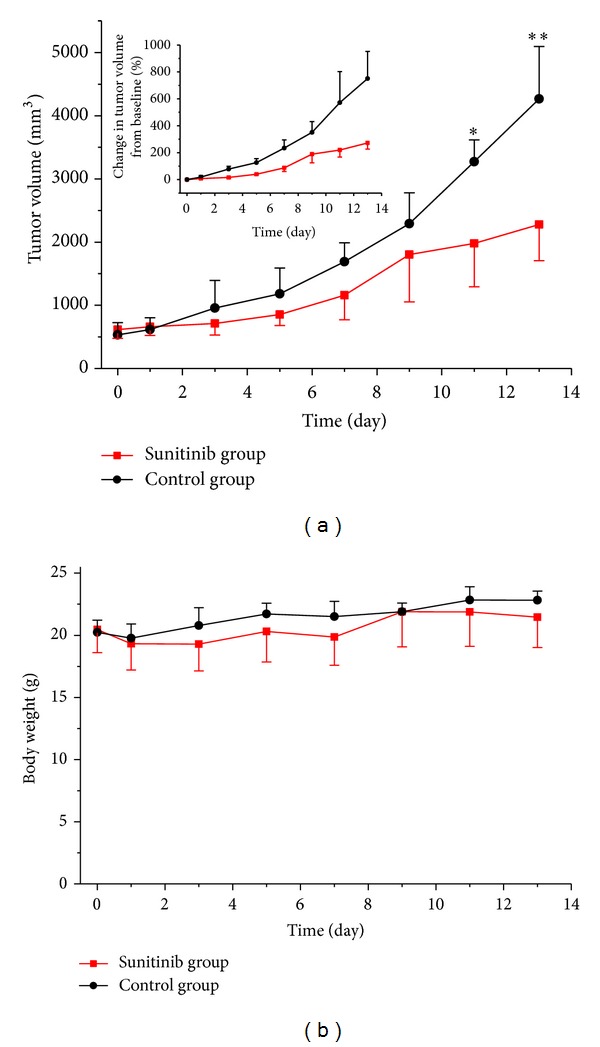
Antitumor activity of Sunitinib in U87MG xenografts. (a) Tumor volume or (V − V_0_)/V_0_ of U87MG tumor-bearing mice treated with vehicle or Sunitinib. There was significant difference for the tumor size between the Sunitinib group and control group after day 11 (*P* = 0.015). (b) Body weight of U87MG tumor-bearing mice treated with vehicle or Sunitinib. There was no difference for the mice weight between the Sunitinib group and control group. *P < 0.05 and **P < 0.01.

As expected, intragastrical administration of consecutive 7 doses of Sunitinib (80 mg/kg) led to a delay in tumor volume. A time-related increase in tumor volume was observed in the control group (Figure 1(a)), in which the average percentage of tumor volume increases, expressed as (V − V_0_)/V_0_, were 18.5 ± 12.2%, 77.9 ± 20.8%, 234.6 ± 60.6%, and 750.6 ± 201.9% on days 1, 3, 7, and 13, respectively. As a comparison, Sunitinib treatment resulted in lower (V − V_0_)/V_0_ of 7.6 ± 4.2%, 15.9 ± 9.1%, 87.0 ± 26.7%, and 272.1 ± 45.9% on days 1, 3, 7, and 13, respectively. There was significant difference for the tumor size between the Sunitinib group and control group after day 11 (*P* < 0.05). In the treated mice, average percentage of tumor volume increase on day 9, (V − V_0_)/V_0_ = 188.8 ± 64.8%, was slightly above the trend line, which may be attributed to the rebound phenomenon that resulted from the sudden stop of Sunitinib (picture inset in Figure 1(a)). This change was also observed in a previous report [23, 24]. Mice body weight was measured as an indicator of the toxic side effects of Sunitinib. As shown in Figure 1(b), no significant body weight loss was observed during the treatment period at the dosage 80 mg/kg of Sunitinib used in this study.

### 3.2. Sunitinib Treatment Inhibited Tumor Cell Proliferation

**Figure 2:**
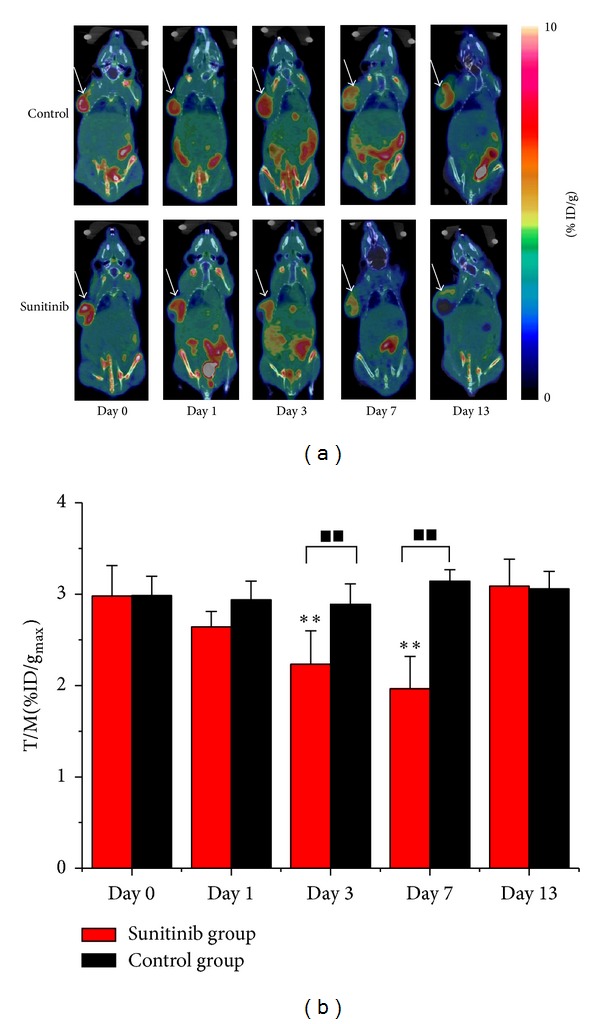
^18^F-FLT microPET/CT imaging of U87MG tumor-bearing mice. (a) Representative decay-corrected whole-body coronal microPET/CT images at 1.0 h after intravenous injection of ^18^F-FLT (11.1 MBq per mouse) on days 0, 1, 3, 7, and 13 after treatment was initiated. (b) The ratios of %ID/g_max⁡_ of tumor to the contralateral muscle (T/M) in the Sunitinib and control groups based on quantitative ROIs analysis from ^18^F-FLT microPET/CT on days 0, 1, 3, 7, and 13 after treatment. The tumors are indicated by* arrows*. **P < 0.01, within the Sunitinib group, compared to day 0. ^■■^ *P* < 0.01, between the Sunitinib group and the control group.

Static microPET/CT scans (Figure 2(a)) at 1.0 h after injection of ^18^F-FLT were acquired on days 0 (baseline), 1, 3, 7, and 13.  Figure 2(b) described the ratios of %ID/g_max⁡_ of tumor to the contralateral muscle (T/M) of ^18^F-FLT in the Sunitinib and control groups. After Sunitinib treatment, the U87MG tumor uptake of ^18^F-FLT decreased from baseline to day 3 (T/M_0_ = 2.98 ± 0.33, T/M_3_ = 2.23 ± 0.36, *P* < 0.001), rapidly reaching the bottom on day 7 when therapy stopped (T/M_7_ = 1.96 ± 0.35, *P* < 0.001), which represented a decrease of up to 34%. On day 13, 6 days after the withdrawal of treatment, ^18^F-FLT uptake recovered (T/M_13_ = 3.09 ± 0.29). Compared to the drastic fluctuations in the Sunitinib group, T/M of ^18^F-FLT uptake in the control group remained around 3.0 throughout the two-week study. Significant differences between the treatment group and the vehicle group were observed on days 3 and 7, where both of *P* values were less than 0.001.

### 3.3. SUV_mean_, SUV_max_, %ID/g_mean_, and %ID/g_max_

**Figure 3:**
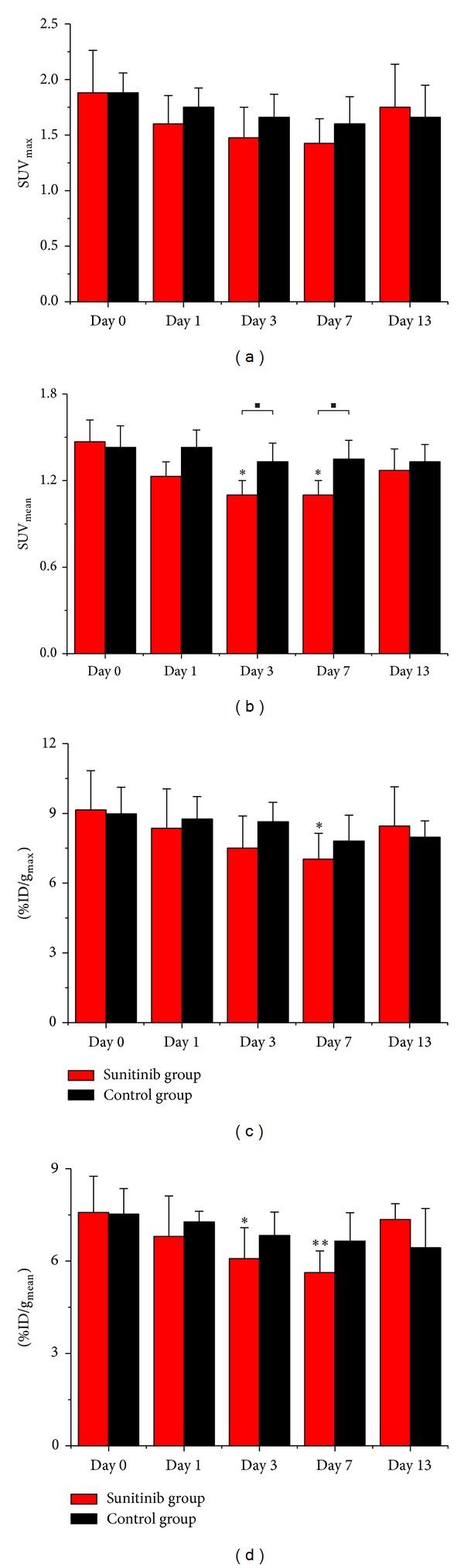
Quantitative ROIs analysis of tumor uptake from ^18^F-FLT microPET/CT. ((a) SUV_max⁡_, (b) SUV_mean_, (c) %ID/g_max⁡_, and (d) %ID/g_mean_) *P < 0.05 and **P < 0.01, within the Sunitinib group, compared to day 0. ^■^ *P* < 0.05, between the Sunitinib group and the control group.

In this study, we compared 5 different parameters (SUV_mean_, SUV_max⁡_, %ID/g_mean_, %ID/g_max⁡_, and T/M) to choose out the most suitable evaluation criterion. No significant differences were observed between the Sunitinib group and the control group for SUV_max⁡_, %ID/g_mean_, and %ID/g_max⁡_, whereas only for SUV_mean_ this was observed on days 3 and 7. Within the Sunitinib group, decreases in SUV_mean_ and %ID/g_mean_ showed statistical differences on days 3 and 7, compared to the baseline (day 0), while decreases in %ID/g_max⁡_ only revealed significant differences on day 3 (Figures 3(a)–3(d)).

### 3.4. Immunohistochemistry and Histology

**Figure 4:**
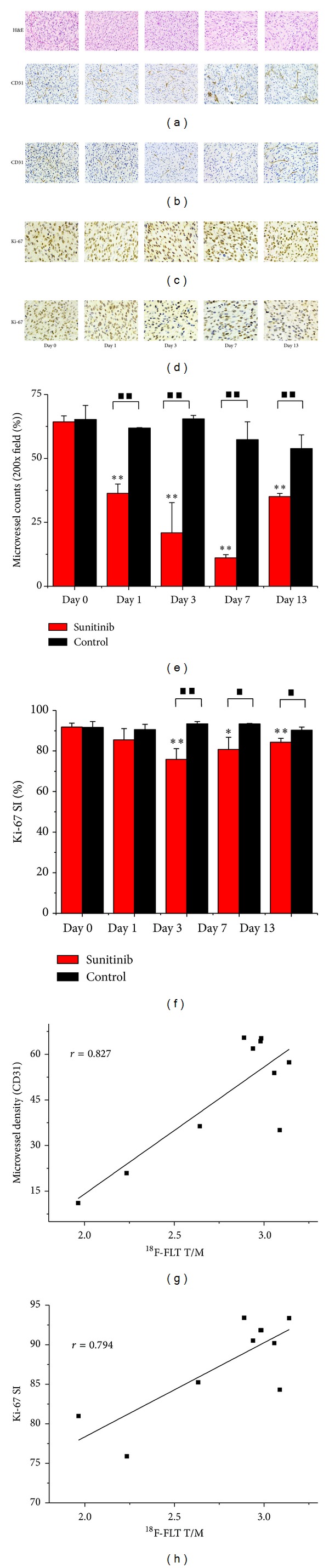
Immunohistochemical and histologic analysis of tumor sections about CD31, Ki67, and H&E on days 0, 1, 3, 7, and 13 after therapy. Top line showed H&E staining in the Sunitinib group. CD31 staining in the Sunitinib group revealed effective antiangiogenic activity from day 1 to day 7 ((a) control, (b) Sunitinib, and 40 × 10, (e)). Ki67 showed a remarkable decrease after the initiation of treatment, which was the most pronounced one on day 3 compared with that for the control tumors ((c) control, (d) Sunitinib, and 40 × 10, (f)). T/M of ^18^F-FLT uptakes of tumor in the treatment group were correlated well with the quantitative data of MVD (g) and Ki67 SI (h). *P < 0.05 and **P < 0.01, within the Sunitinib group, compared to day 0. ^■^ *P* < 0.05 and ^■■^ *P* < 0.01, between the Sunitinib group and the control group.

Figure 4 shows representative tumor sections of haematoxylin and eosin (H&E), CD31, and Ki-67 staining for the control and Sunitinib groups on days 0, 1, 3, 7, and 13 after therapy. CD31-positive staining was broadly observed in all untreated tumor sections, which demonstrated relatively abundant microvessel density (MVD). After Sunitinib treatment, the MVD level in tumor sections decreased remarkably, which indicated effective antiangiogenic activity of the drug. The MVD level lowered to 36.33 ± 3.64% on day 1 after therapy, compared with that of the vehicle group (61.88 ± 0.16, *P* = 0.003) and continuously declined until day 7 (*P* < 0.001) (Figure 4(e)). The Ki-67 SI showed a remarkable decrease after the initiation of treatment, which was the most pronounced one on day 3 compared with that for the control tumors (*P* = 0.005). On day 13, the Ki-67 SI returned yet to be still less than the baseline level. The untreated tumors remained with a relatively high proliferation rate with Ki67 SI of more than 90% (Figure 4(f)). T/M of ^18^F-FLT uptakes were correlated well with the quantitative data of MVD and Ki-67 SI (Figures 4(g)-4(h)).

## 4. Discussion

Angiogenesis, the formation of new blood vessels, has been proved to be critical in the growth and invasiveness of solid tumors, which makes antiangiogenesis become the widely used targeted therapy of cancer up to today [25]. Antiangiogenic agents include tyrosine kinase inhibitors (TKI) like Sunitinib and vascular endothelial growth factor- (VEGF-) targeted antibody Bevacizumab. Sunitinib is a multitargeted TKI which results in VEGF signaling blockade to suppress cancer cell growth. Thus, compared with the conventional cytotoxic agents, TKI may provide a more tolerable cytostatic therapy against solid tumors with diverse histology, either as monotherapy or in combination with radiation and/or additional chemotherapy [26]. Various TKI agents, such as SU11248 (Sunitinib), Bevacizumab, and GW786034 (Pazopanib), have been approved by FDA for clinical applications [27–30]. Single-agent TKIs have low objective rates of response, which are usually less than 50% in the selected patients [10, 31]. Therefore, noninvasive visualization and quantification of antitumor and antiangiogenesis potency would be of importance for patient selection, dosage optimization, and dose intervals of compounds in this category [16]. Herein, we aimed to monitor antiangiogenic treatment response of Sunitinib in U87MG tumor xenografts mimicking GBM by dynamic ^18^F-FLT microPET/CT imaging in this study.

Our longitudinal study proved the value of quantitative ^18^F-FLT PET/CT imaging in monitoring early response to the antiangiogenic therapy (ATT) of Sunitinib in U87MG tumor xenografts. In the Sunitinib treatment group, the ratio of %ID/g_max⁡_ of tumor to the contralateral muscle (T/M) of ^18^F-FLT decreased by 25% on day 3 and by 34% on day 7, whereas T/M in the control group remained around the baseline level throughout the study. It was beyond our expectation that another 4 parameters (SUV_max⁡_, SUV_mean_, %ID/g_mean_, and %ID/g_max⁡_) had not shown statistical value in this study. They were often used to evaluate therapy response in previous studies and gave rise to controversial results [14–16, 23], because they as single measured values (SUV_mean_, SUV_max⁡_, %ID/g_mean_, and %ID/g_max⁡_) could be influenced by various factors such as individual difference among mice, change of physical state, operation errors, and therapeutic intervention, even to the utmost extent avoiding the impact of those factors. It might remind us that the parameters beyond SUV and %ID/g could be of benefit to response evaluation. Significant difference for the tumor size between the Sunitinib group and control group was observed until day 11 according to the traditional RECIST. Quantitative ^18^F-FLT PET/CT imaging can not only reflect the status of tumor cell proliferation but also distinguish residual viable tumor tissue from fibrosis [13]. For example, Figure 2(a) showed a large necrosis area without ^18^F-FLT uptake in Sunitinib-treated tumors on day 13. Although tumor sizes in untreated group were larger than that in Sunitinib-treated group, it yet displayed uniform tumor uptake of ^18^F-FLT without remarkable necrosis area, which further illustrated the ATT effects of Sunitinib. This was also validated by the decrease of Ki67 SI and MVD after therapy initiation.

There have been several reports about the use of various PET tracers to predict ATT efficacy of TKIs in solid tumors [16, 23, 32, 33]. For example, Battle et al. used ^18^F-Fluciclatide to detect the therapeutic response after a 2-week dosing regimen (60 mg/kg) of Sunitinib in U87MG tumors [23]. They found that the uptake level of ^18^F-Fluciclatide in tumor sites from the Sunitinib-treated group immediately decreased on day 2, and a level of MVD expression was observed on day 13 significantly lower than that in control animals. However, ^18^F-Fluciclatide as a new PET biomarker is without easy accessibility at present and another limitation of their study was not to compare longitudinal MVD level due to the lack of histopathologic data from tumor samples at early time points. Morrison et al. applied ^18^F-AH111585 to monitor ZD4190 therapy response of Calu-6 nonsmall cell lung tumor xenografts [32]. A significant decrease (31.8%) in ^18^F-AH111585 uptake was discovered, which proved that ^18^F-labeled RGD tracer could noninvasively monitor the antiangiogenic effect of ZD41190. Yang et al. established MDA-MB-435 breast cancer xenografts and evaluated the early response to ZD4190 by ^18^F-FDG, ^18^F-FLT, and ^18^F-FPPRGD2 [16]. In ZD4190-treated tumors, the uptake of ^18^F-FLT decreased by 8.1% on day 1 and by 21% on day 3, which was confirmed by Ki67 SI. However, ^18^F-FDG uptake in tumors with or without treatment showed no significant difference, even increasing slightly in ZD4190-treated tumors on day 3. The uptake of ^18^F-FDG in inflammatory cells may lead to an overestimation of the viable tumor cells. In contrast, ^18^F-FLT uptake would be less disturbed by the inflammatory response because inflammatory cells have only minor proliferation tendency [34]. They found that ^18^F-FPPRGD2 PET/CT imaging showed lower background and higher tumor/muscle ratio compared with ^18^F-FLT imaging, and the magnitude of the changes in tumor uptake of ^18^F-FPPRGD2 was also higher than that of ^18^F-FLT. As a result, they considered that ^18^F-FPPRGD2 would be superior to ^18^F-FLT as a PET probe for predicting the early response of tumor to ZD4190. Nevertheless, ^18^F-FPPRGD2 has not been put into the clinical application due to its complicated synthesis steps and relatively low radiochemical yield. ^18^F-Fluciclatide and ^18^F-AH111585 faced also the same kind of limitations as those of ^18^F-FPPRGD2 under current conditions. Therefore, ^18^F-FLT, a well-established PET molecular imaging probe, was selected to monitor treatment response of Sunitinib in U87MG xenografts in our study. At last, our results proved the great potential value of ^18^F-FLT PET/CT imaging protocol for monitoring the early response of Sunitinib.

## 5. Conclusions

We investigated the feasibility of longitudinal ^18^F-FLT PET/CT to monitor the early response of antiangiogenesis therapy of Sunitinib in U87MG xenografts. This protocol could be easily translated into clinical trials and make contribution to treatment plan of antiangiogenesis treatment in the future.

## Supplementary Material

The following two figures are representative IHC captures about CD31 on day 0 and 3 after Sunitinib treatment.

## Figures and Tables

**Table 1:** Experimental design for longitudinal ^18^F-FLT microPET/CT imaging of Sunitinib treatment efficacy.

Parameter	Day								
	0	1	2	3	4	5	6	7	13
^18^F-FLT									
Sunitinib	*✓*	+ *✓*	+	+ *✓*	+	+	+	+ *✓*	*✓*
Control	*✓*	+ *✓*	+	+ *✓*	+	+	+	+ *✓*	*✓*
Histology									
Sunitinib	×	+ ×	+	+ ×	+	+	+	+ ×	×
Control	×	+ ×	+	+ ×	+	+	+	+ ×	×

*✓*: microPET/CT; +: vehicle or Sunitinib treatment; ×: tumor sampling.

## Abbreviations

GBM: Glioblastoma multiforme
ATT: Antiangiogenic therapy
TKIs: Tyrosine kinase inhibitors
RECIST: Response evaluation criteria in solid tumors
PERCIST: PET response evaluation criteria in solid tumors
VEGF: Vascular endothelial growth factor
MVD: Microvessel density
SPSS: Statistical package for the social science.
