# Supplementary figures and images for: Early Monitoring Antiangiogenesis Treatment Response of Sunitinib in U87MG Tumor Xenograft by 18F-FLT MicroPET/CT Imaging
Source: Biomed Res Int. 2014 Apr 9;2014:218578. doi: 10.1155/2014/218578 (PMC4000939; doi:10.1155/2014/218578)

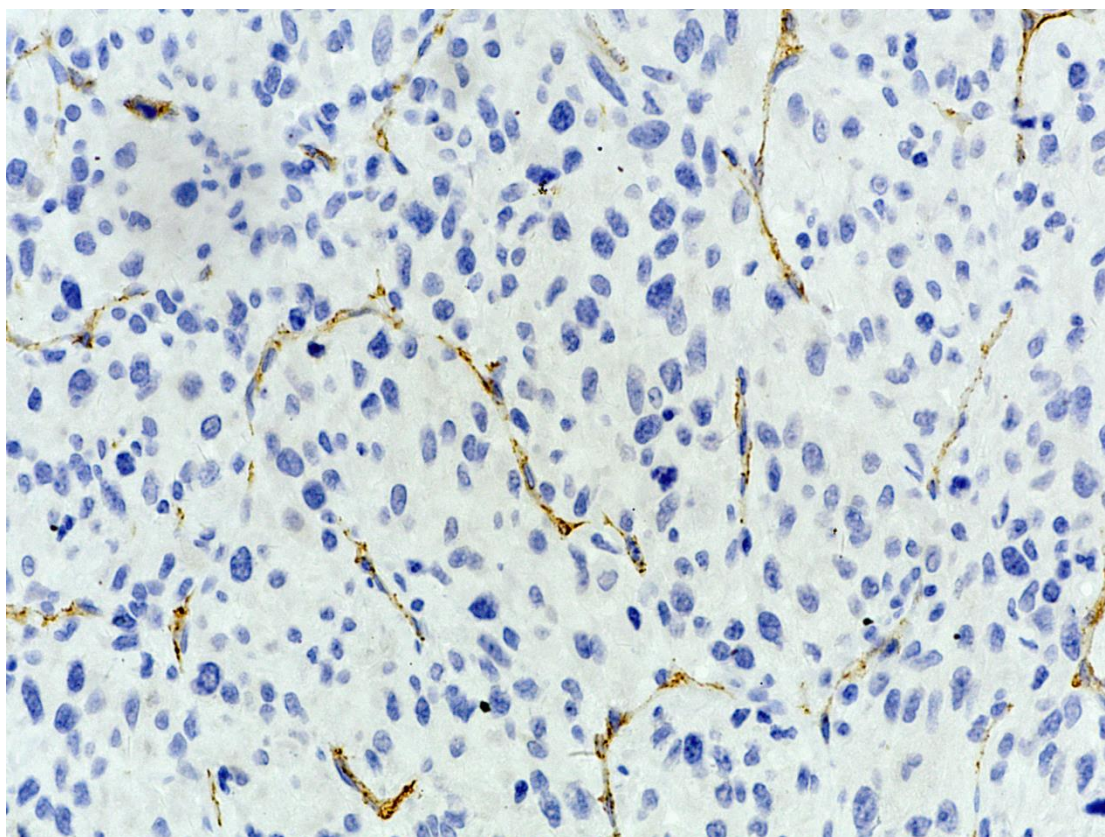

**Sunitinib-Day0**

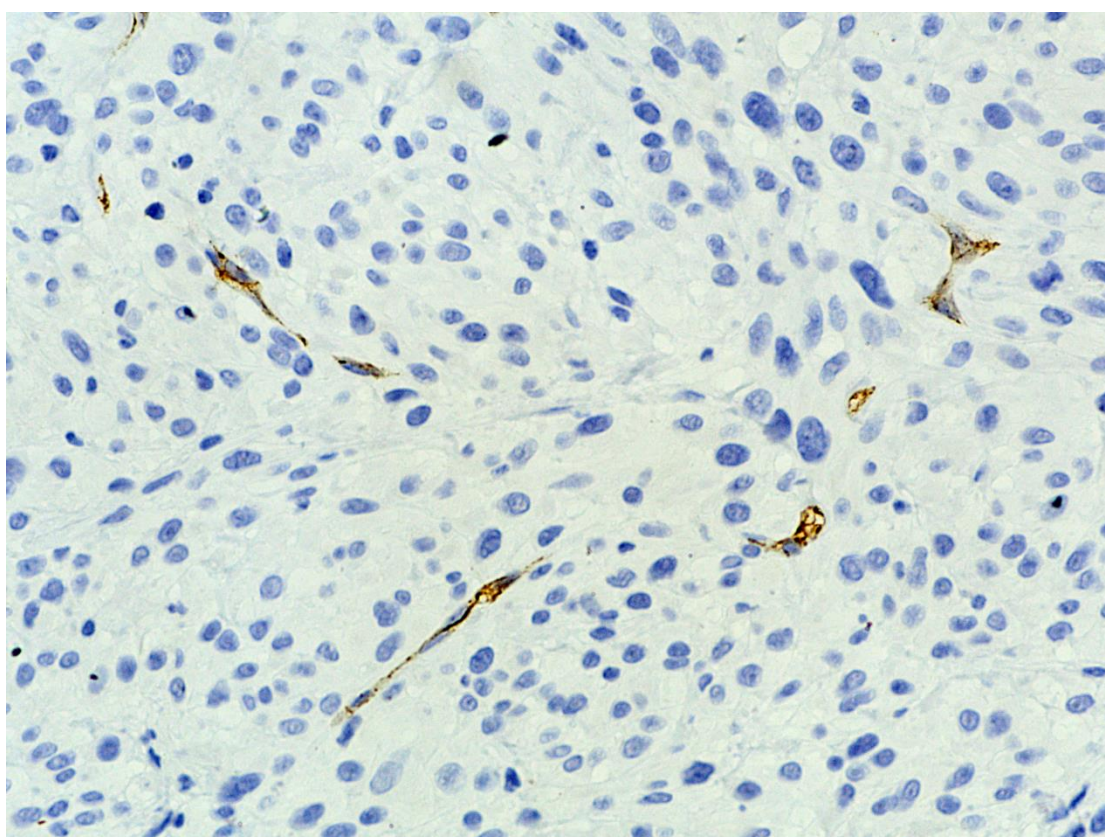

## Sunitinib-Day3

Supplement: Supplementary file 1 — The following two figures are representative IHC captures about CD31 on day 0 and 3 after Sunitinib treatment. [file 218578.f1.pdf]
